# Fatores individuais, familiares e comunitários associados ao uso de contracepção de emergência por adolescentes escolares brasileiros

**DOI:** 10.1590/0102-311XPT148323

**Published:** 2024-11-25

**Authors:** Marco Aurélio de Sousa, Luana Leão Menezes, Ed Wilson Rodrigues Vieira, Gisele Nepomuceno de Andrade, Deborah Carvalho Malta, Mariana Santos Felisbino-Mendes

**Affiliations:** 1 Programa de Pós-graduação em Enfermagem, Universidade Federal de Minas Gerais, Belo Horizonte, Brasil.; 2 Escola de Enfermagem, Universidade Federal de Minas Gerais, Belo Horizonte, Brasil.

**Keywords:** Adolescente, Saúde Reprodutiva, Anticoncepção, Anticoncepcionais Pós-coito, Contracepção Hormonal, Adolescent, Reproductive Health, Contraception, Postcoital Contraceptives, Hormonal Contraception, Adolescente, Salud Reproductiva, Anticoncepción, Anticonceptivos Poscoito, Anticoncepción Hormonal

## Abstract

O objetivo foi estimar a proporção do uso de contracepção de emergência alguma vez na vida entre adolescentes escolares brasileiros e a associação de fatores individuais, familiares e comunitários com o uso. Realizou-se estudo transversal, com amostra de 38.779 adolescentes escolares brasileiros, de 13 a 17 anos, respondentes da *Pesquisa Nacional de Saúde do Escolar* (PeNSE) em 2019 que declararam iniciação sexual. Estimou-se a proporção do uso de contracepção de emergência alguma vez na vida e a forma de acesso ao método. As razões de proporções foram estimadas para avaliar quais os fatores associados ao uso de contracepção de emergência alguma vez na vida. A proporção de adolescentes que usou contracepção de emergência, ou que informaram o uso por seus parceiros, alguma vez na vida foi 37,9%. Quanto ao acesso à contracepção de emergência, as farmácias comerciais foram a principal forma de aquisição. Os adolescentes de 16 e 17 anos, os que procuraram por serviço de saúde no último ano, os residentes nas regiões Centro-oeste e Sudeste, e os com histórico de violência sexual usaram contracepção de emergência ou informaram o uso alguma vez na vida por seus parceiros com maior frequência. Residir na Região Sul do país esteve associado à menor proporção de adolescentes que usaram contracepção de emergência ou que informaram o uso por parceiros alguma vez na vida. A associação com fatores individuais, familiares e comunitários relacionados ao uso de contracepção de emergência pode refletir a não utilização ou falhas no uso de outros métodos contraceptivos, revelando lacunas nas políticas públicas relacionadas à saúde sexual e reprodutiva dos adolescentes no país.

## Abstract (en)

This study aimed to estimate the proportion of use of emergency contraception at least once in life among Brazilian adolescent students, as well as the association of individual, family, and community factors with use. A cross-sectional study was carried out, including 38,779 Brazilian adolescent students, aged 13 to 17 years, participating in the *Brazilian National Survey of School Health* (PeNSE) in 2019, who declared sexual initiation. The proportion of emergency contraception use at least once in life and the form of access to the method were estimated. Proportion ratios were estimated to assess the factors associated with the use of emergency contraception at some point in life. The proportion of adolescents who used emergency contraception, or who reported the use by their partners, at some point in their lives was 37.9%. Regarding the access to the emergency contraception, commercial pharmacies were the main source of purchase. Adolescents aged 16 and 17 years, those who sought health services in the last year, those living in the Central-West and Southeast regions, and those who had a history of sexual violence used emergency contraception or reported their partners using them at least once in their lives. Living in the South region of Brazil was associated with a lower proportion of adolescents who used emergency contraception or who reported the use by their partners at least once in their lives. The association with individual, family, and community factors related to the use of emergency contraception may reflect the non-use or failures in the use of other contraceptive methods, revealing gaps in public policies related to the sexual and reproductive health of adolescents in Brazil.

## Resumen

El objetivo fue estimar la proporción de uso de anticoncepción de emergencia alguna vez entre adolescentes escolares brasileños y la asociación de factores individuales, familiares y comunitarios con su uso. Se realizó un estudio transversal, con una muestra de 38.779 adolescentes escolares brasileños, de 13 a 17 años, entrevistados en la *Encuesta Nacional de Salud del Escolar* (PeNSE) en el 2019 que declararon iniciación sexual. Se estimó la proporción de uso de anticoncepción de emergencia alguna vez en la vida y la forma de acceder al método. Se estimaron ratios de proporciones para evaluar qué factores estaban asociados con el uso de anticoncepción de emergencia en algún momento de la vida. La proporción de adolescentes que usaron anticoncepción de emergencia, o que reportaron el uso por parte de sus parejas, en algún momento de sus vidas fue del 37,9%. En cuanto al acceso a la anticoncepción de emergencia, las farmacias comerciales fueron la principal forma de adquisición. Los adolescentes de 16 y 17 años, los que buscaron servicios de salud en el último año, los residentes en las regiones Centro-Oeste y Sudeste y aquellos con antecedentes de violencia sexual utilizaron anticoncepción de emergencia o reportaron el uso por parte de sus parejas en algún momento de sus vidas con más frecuencia. Vivir en la región Sur del país se asoció con una menor proporción de adolescentes que utilizaron anticoncepción de emergencia o que reportaron el uso por parte de sus parejas en algún momento de sus vidas. La asociación con factores individuales, familiares y comunitarios relacionados con el uso de anticoncepción de emergencia puede reflejar la falta de uso o las fallas en el uso de otros métodos anticonceptivos, lo que revela brechas en las políticas públicas relacionadas con la salud sexual y reproductiva de los adolescentes en el país.

## Introdução

O início da vida sexual geralmente ocorre na adolescência. Cerca de um quarto dos brasileiros de 13 a 15 anos, e seis em cada dez entre 16 a 17 anos, já iniciaram sua vida sexual ^1^. Desses, aproximadamente 40% não utilizaram método contraceptivo na última relação sexual ^1^, corroborando outros estudos ^2^ ^,^ ^3^ ^,^ ^4^ ^,^ ^5^ ^,^ ^6^ que confirmam a baixa prevalência do uso de métodos contraceptivos nesse grupo.

O uso de contracepção por adolescentes constitui-se em um direito sexual e reprodutivo, necessário para prevenção de gravidez, além de ser indicador de acesso desse grupo aos métodos contraceptivos. Uma gravidez não planejada na adolescência gera repercussões negativas, tanto relacionadas às condições econômicas e sociais ^7^ ^,^ ^8^ ^,^ ^9^ ^,^ ^10^ quanto à saúde ^7^ ^,^ ^11^ e à educação, com destaque para a evasão escolar ^12^. E, para que a contracepção seja eficaz, é preciso garantir a provisão de métodos contraceptivos modernos e seguros ^6^ ^,^ ^9^.

A inclusão de contraceptivos de emergência, também conhecidos como pílulas do dia seguinte, entre os métodos contraceptivos por parte do Ministério da Saúde brasileiro deu-se, de forma mais ampla a partir de 2003, com a Política Nacional de Atenção Integral à Saúde da Mulher ^12^, representando uma das importantes conquistas da luta pelos direitos sexuais e reprodutivos no país ^13^. Essa contracepção tem como objetivo evitar a gravidez após relação sexual desprotegida ^9^. No Brasil, a pílula de levonorgestrel é a mais utilizada e mais eficaz para esses fins ^9^ ^,^ ^14^, com distribuição gratuita pelo Sistema Único de Saúde (SUS) ^14^.

O prazo recomendado para uso de contraceptivos de emergência é de até 120 horas após a relação sexual, e sua maior eficácia está relacionada ao uso precoce. Estudo avaliou o desconforto de pediatras em prescreverem contraceptivos de emergência para adolescentes e mostrou que apenas 15,2% desses profissionais tinham conhecimento sobre esse tempo máximo ^15^.

O uso de contraceptivos de emergência é comum entre adolescentes ^16^, muitas vezes devido a fragilidades no campo da saúde sexual e reprodutiva desse grupo ^17^. Estudo apontou que um terço dos adolescentes sexualmente ativos já utilizaram esse método alguma vez na vida ^6^, enquanto outro apontou que apenas 10% fizeram uso no último ano ^5^. Mais da metade das adolescentes sexualmente ativas das regiões Sudeste e Sul do Brasil já fizeram uso de contraceptivos de emergência alguma vez na vida ^18^. Desse modo, observa-se variação entre os achados em relação à proporção do uso de contraceptivos de emergência, o que pode ser justificado pelas diferentes populações e amostras estudadas, de âmbito local e regional, às vezes somente de meninas, mas que em todas demonstram magnitude relevante o suficiente para a atenção das autoridades em saúde sobre o uso de contraceptivos de emergência.

Estudos demonstraram que grande parte dos adolescentes tinham algum tipo de conhecimento acerca da contracepção de emergência ^5^ ^,^ ^6^, porém são poucos os que investigaram o uso em amostras abrangentes de adolescentes brasileiros. Nas regiões Sudeste e Sul, observa-se que mais de 90% das adolescentes conhecem a contracepção de emergência, e, geralmente, as informações são adquiridas com amigos ^18^. Além disso, a maior parte dos estudos incluem universitários, nos quais aproximadamente metade já utilizaram contraceptivos de emergência alguma vez na vida ^19^ ^,^ ^20^; porém, nesse grupo, estão também os adultos jovens.

Até o momento, não se sabe ao certo a magnitude nacional do uso de contraceptivos de emergência por adolescentes, bem como aspectos relacionados ao acesso ao método, visto que fatores individuais, familiares e comunitários, tais como sexo, escolaridade da mãe e residir em áreas urbanas ou rurais, podem estar associados ao maior ou menor uso da contracepção de emergência ^21^, conforme modelo socioecológico de uso de contracepção por adolescentes ^21^. Pode-se, ainda, inferir possíveis desigualdades nesse acesso no contexto brasileiro, em relação às regiões do país, sexo e faixa etária. Ademais, alguns estudos têm apontado barreiras ao acesso à contracepção de emergência ^22^, tais como baixas condições socioeconômicas das mulheres ^9^, o que também poderia se aplicar à realidade do adolescente. Assim, tem-se como hipótese que fatores individuais, familiares e comunitários estão associados ao uso de contraceptivos de emergência por adolescentes escolares brasileiros.

Desse modo, este estudo tem por objetivos estimar a proporção do uso de contracepção de emergência alguma vez na vida entre adolescentes escolares brasileiros e a associação de fatores individuais, familiares e comunitários com o uso.

## Métodos

### Desenho e população de estudo

Trata-se de estudo transversal que utilizou dados de adolescentes escolares brasileiros respondentes da *Pesquisa Nacional de Saúde do Escolar* (PeNSE), realizada em 2019. A pesquisa incluiu uma amostra de 125.123 adolescentes, representativa dos adolescentes brasileiros de 13 a 17 anos, matriculados do 7º ao 9º ano do Ensino Fundamental ou do 1º ao 3º ano do Ensino Médio em escolas públicas ou privadas ^23^. As perguntas relacionadas ao uso de métodos contraceptivos foram feitas somente aos 38.779 adolescentes da amostra que reportaram que já haviam tido relação sexual (21.966 meninos e 16.687 meninas). Para as perguntas sobre a contracepção de emergência, os meninos responderam considerando o uso por suas parceiras. Maior detalhamento do plano amostral da PeNSE pode ser consultado em: https://bit.ly/3ONZEwq.

### Coleta de dados

A coleta de dados da PeNSE 2019 foi realizada por questionário estruturado e autoaplicável, utilizando dispositivos móveis/*smartphones* ^23^. O questionário tinha 14 blocos temáticos, incluindo o de saúde sexual e reprodutiva, de interesse neste estudo. O bloco saúde sexual e reprodutiva apresentava 13 perguntas, duas delas sobre o uso de contracepção de emergência.

### Variáveis de interesse

As perguntas para avaliar o uso de contracepção de emergência foram: (1) “Alguma vez na vida, você ou sua parceira já usou pílula do dia seguinte (contracepção de emergência)?”, com as respostas: Sim e Não; e (2) “Na última vez que você ou sua parceira usou pílula do dia seguinte (contracepção de emergência) como conseguiu?”, com as opções de respostas: No serviço de saúde, Com um(a) amigo(a) ou colega, Com mãe, pai ou responsável, Comprei na farmácia, Com o(a) parceiro(a) sexual e Com outra pessoa ou de outro modo.

Foram considerados como desfechos: (1) uso de contraceptivos de emergência pelas adolescentes alguma vez na vida e (2) acesso à contracepção de emergência (modos de ter acesso ao método). As variáveis explicativas foram organizadas de acordo com os níveis individuais, familiares e comunitários, conforme modelo socioecológico desenvolvido por Bronfenbrenner ^24^ para avaliar o desenvolvimento infantil e posteriormente adaptado para estudo dos fatores que se relacionam à saúde dos adolescentes, incluindo os desfechos de saúde sexual e reprodutiva ^21^.

Como fatores individuais, foram incluídas as variáveis: sexo (masculino e feminino); faixas etárias em anos (13 a 15 e 16 e 17); raça ou cor (branca, preta, parda, amarela e indígena); histórico de gravidez (sim e não); procura por serviço de saúde no último ano (sim e não); procura por unidade básica de saúde (UBS) no último ano (sim e não); vacinação contra o HPV (sim e não). Também foi levado em consideração os históricos de violência doméstica (sim e não); de importunação sexual (sim e não); e de violência sexual (sim e não).

Para os fatores familiares, foram consideradas: escolaridade da mãe (não estudou, Ensino Fundamental, Ensino Médio ou Ensino Superior) e morar com pais (com ambos os pais, somente com a mãe, somente com o pai ou com nenhum dos pais). Além disso, com base em estudos prévios ^25^ ^,^ ^26^ ^,^ ^27^, utilizamos um escore de supervisão dos pais a partir das variáveis falta de aula sem permissão dos pais, pais cientes das atividades em tempo livre, pais entendem problemas e preocupações e pais presentes nas refeições. Esse escore varia de 0, nenhuma supervisão, a 4, intensa supervisão: 0 quando resposta sim para a variável que analisa a falta às aulas sem a permissão dos pais e respostas não para as outras variáveis; 4 quando resposta não para a variável que analisa a falta às aulas dos adolescentes sem a permissão dos pais e respostas sim para as outras variáveis ^25^. Trata-se de medida *proxy* de monitoramento dos pais no sentido de que práticas familiares como a supervisão e presença dos pais podem se constituir em fator protetor para comportamentos em saúde ^25^ ^,^ ^28^.

Em relação aos fatores comunitários considerou-se: região (Norte, Nordeste, Sudeste, Sul, Centro-oeste); área de moradia (rural ou urbana); dependência administrativa da escola (pública e privada); orientação sobre prevenção de gravidez na escola (sim e não); sobre prevenção de infecções sexualmente transmissíveis (IST) (sim e não); e sobre acesso ao preservativo gratuito (sim e não).

### Análise de dados

Primeiramente, estimaram-se as proporções do uso de contraceptivos de emergência entre as adolescentes que já haviam tido relação sexual alguma vez na vida e dos modos de acesso ao método, com os respectivos intervalos de 95% de confiança (IC95%) por sexo, idade, região, Unidade da Federação (UF) e dependência administrativa da escola do adolescente entrevistado. Em seguida, procedeu-se com análise univariada entre cada variável explicativa e o desfecho, com estimativas da razão de proporções (RP) não ajustada por meio da regressão binomial negativa. Estimou-se as RP ajustadas com a modelagem multivariada para a qual foram consideradas as variáveis explicativas que na univariada tiveram valor de p < 0,200. Procedeu-se com a entrada das variáveis uma a uma por meio do critério *forward*, utilizando ordem de entrada primeiro dos fatores mais proximais (individuais), seguidos dos intermediários (familiares) e por último dos fatores mais distais (comunitários). As variáveis com valor de p > 0,05 foram retiradas do modelo, utilizando-se o critério *backward*. Utilizou-se o teste de Wald em cada entrada de variável no modelo e após o modelo final, para avaliar a contribuição de cada variável na modelagem ^29^. A relação entre as variáveis explicativas também foi avaliada para inserção no modelo multivariado, por meio da distribuição entre cada duas variáveis e o teste qui-quadrado de Pearson. A partir dessa análise, observou-se que aqueles que receberam orientação sobre prevenção de gravidez em sua maioria também receberam sobre acesso ao preservativo (68,6%) e prevenção de IST (75,2%) (p < 0,0001). Isso também foi observado entre morar com os pais e supervisão dos pais, de forma moderada (58%). Adicionalmente, as variáveis explicativas com baixa taxa de resposta (vacina HPV) ou respondidas somente pelas meninas (histórico de gravidez) não entraram no modelo final. Foi usado o software Stata, versão 14.0 (https://www.stata.com), no módulo *survey*. A base de dados foi extraída em: https://bit.ly/3BZvq27.

### Considerações éticas

A PeNSE 2019 foi aprovada pela Comissão Nacional de Ética em Pesquisa (CONEP; parecer nº 3.249.268, de 8 de abril de 2019). Os participantes precisaram concordar com o Termo de Consentimento Livre e Esclarecido e a abertura do questionário estava vinculada ao aceite. A participação foi voluntária e os adolescentes poderiam deixar de responder qualquer questão ou abandonar o questionário a qualquer momento ^23^.

## Resultados

Dos 33.711 adolescentes que declararam iniciação sexual: 50,3% (IC95%: 49,0-51,5) era do sexo masculino; 64,1% (IC95%: 62,9-65,3) se autodeclararam pretos e pardos; 35% (IC95%: 33,8-36,3) tinham mãe que estudaram até o Ensino Médio; 46,9% (IC95%: 45,6-48,1) moravam com ambos os pais; 39% (IC95%: 37,5-40,6) residia na Região Sudeste; e 90,4% (IC95%: 90,0-90,8) estudavam em escola pública (Tabela 1).

**Tabela 1:** Características sociodemográficas e familiares dos adolescentes escolares que tiveram iniciação sexual e responderam sobre o uso de contracepção de emergência. *Pesquisa Nacional de Saúde do Escolar* (PeNSE), 2019, Brasil (n = 33.711).

Variáveis sociodemográficas e familiares	% * (IC95%)
Sexo	
Feminino	49,7 (48,4-51,0)
Masculino	50,3 (49,0-51,5)
Região de moradia	
Norte	12,3 (11,6-12,9)
Nordeste	26,5 (25,4-27,6)
Sudeste	39,0 (37,5-40,6)
Sul	13,9 (13,1-14,8)
Centro-oeste	8,3 (7,9-8,8)
Raça/Cor	
Preta	15,7 (14,8-16,6)
Parda	44,1 (42,9-45,2)
Branca	33,6 (32,5-34,7)
Amarela	3,7 (3,3-4,1)
Indígena	3,0 (2,6-3,4)
Escolaridade da mãe	
Não estudou	5,8 (5,2-6,5)
Ensino Fundamental	33,8 (32,5-35,1)
Ensino Médio	35,0 (33,8-36,3)
Ensino Superior	25,5 (24,3-26,7)
Dependência administrativa da escola	
Pública	90,4 (90,0-90,8)
Privada	9,6 (9,2-10,1)
Reside com os pais	
Com ambos os pais	46,9 (45,6-48,1)
Apenas com a mãe	36,6 (35,6-37,6)
Apenas com o pai	6,3 (5,7-6,8)
Não reside com os pais	10,3 (9,7-11,0)

IC95%: intervalo de 95% de confiança.

* Estimativa populacional.

O uso de contraceptivos de emergência alguma vez na vida foi 37,9% (Tabela 2). A proporção de meninas que responderam ter usado alguma vez na vida foi de 47,1% (39,2% entre as com 13 a 15 anos de idade e 52,3% entre as com 16 e 17). Entre os meninos, 28,8% responderam que suas parceiras já haviam usado (21,5% entre os com 13 a 15 anos de idade e 34,7% entre os de 16 e 17).

**Tabela 2:** Proporção e intervalos de 95% de confiança (IC95%) dos adolescentes escolares brasileiros que já usaram contracepção de emergência alguma vez na vida segundo sexo, faixa etária e região do país. *Pesquisa Nacional de Saúde do Escolar* (PeNSE) 2019, Brasil.

Faixa etária (anos)/Sexo	Brasil	Região				
	Total	Norte	Nordeste	Sudeste	Sul	Centro-oeste
	% (IC95%)	% (IC95%)	% (IC95%)	% (IC95%)	% (IC95%)	% (IC95%)
13 a 15						
Todos	29,8 (28,4-31,2)	31,5 (28,7-34,5)	25,7 (23,5-28,1)	32,9 (30,3-35,6)	24,8 (21,4-28,5)	33,5 (30,5-36,7)
Feminino	39,2 (36,9-41,6)	39,8 (35,1-44,7)	35,5 (31,6-39,6)	44,4 (40,0-49,1)	29,8 (24,9-35,4)	41,8 (37,6-46,3)
Masculino	21,4 (19,7-23,3)	25,4 (21,1-30,3)	17,5 (15,0-20,5)	22,7 (19,5-26,3)	19,1 (15,0-24,2)	25,4 (22,0-29,3)
16 e 17						
Todos	43,8 (42,3-45,3)	40,6 (37,7-43,6)	40,7 (38,2-43,3)	47,5 (44,8-50,2)	39,1 (35,1-43,3)	48,6 (45,6-51,7)
Feminino	52,3 (50,0-54,6)	50,2 (46,6-53,8)	51,3 (47,7-54,9)	56,1 (51,6-60,5)	42,4 (37,2-47,8)	57,6 (53,2-61,4)
Masculino	34,7 (32,8-36,6)	30,2 (26,70-33,9)	30,6 (28,3-32,9)	37,7 (33,8-41,6)	35,2 (30,1-40,7)	39,9 (36,4-43,6)
13 a 17						
Todos	37,9 (36,8-39,0)	36,6 (34,5-38,8)	34,3 (32,3-36,3)	41,4 (39,3-43,5)	33,4 (30,5-36,4)	42,3 (39,8-44,8)
Feminino	47,1 (45,3-48,9)	46,1 (43,0-49,1)	44,8 (41,8-47,9)	51,6 (48,1-55,1)	37,5 (33,7-41,4)	50,9 (47,5-54,2)
Masculino	28,8 (27,4-30,2)	27,8 (25,0-30,8)	24,8 (23,1-26,6)	30,9 (28,1-33,8)	28,8 (25,3-32,5)	33,8 (31,2-36,5)

Entre as meninas do grupo mais jovem, a proporção que respondeu já ter usado foi menor na Região Sul (29,9%), seguido das regiões Nordeste (35,5%), Norte (39,8%), Centro-oeste (41,9%) e Sudeste (44,5%) (Tabela 2). Já entre as mais velhas, a proporção que respondeu já ter usado foi menor entre as que viviam na Região Sul (42,4%), seguidas das que viviam na Norte (50,2%), Nordeste (51,3%), Sudeste (56,1%) e Centro-oeste (57,4%). Entre os meninos mais velhos, observou-se uma menor diferença entre as proporções que responderam ao histórico de uso por suas parceiras em cada região.

Em relação às estimativas por UFs, entre adolescentes de 13 a 17 anos, de ambos os sexos, verificou-se que o Maranhão foi a UF que apresentou a menor proporção de adolescentes que responderam que já usaram contraceptivos de emergência alguma vez na vida (28,5%), seguido do Amazonas (30,6%) e do Rio Grande do Sul (32,7%) (Tabela 3). Entre as que apresentaram as maiores proporções de adolescentes que responderam histórico de uso estão Goiás (44,2%), São Paulo (43%) e o Distrito Federal (42,9%).

**Tabela 3:** Proporção de adolescentes escolares brasileiros de 13 a 17 que já usaram contracepção de emergência alguma vez na vida segundo sexo e Unidade da Federação (UF). *Pesquisa Nacional de Saúde do Escolar* (PeNSE) 2019, Brasil.

UF	Todos	Meninas	Meninos
	% (IC95%)	% (IC95%)	% (IC95%)
Rondônia	40,8 (36,9-44,8)	53,0 (47,9-58,1)	27,8 (22,7-33,5)
Acre	36,4 (33,0-39,9)	44,9 (39,8-49,9)	28,3 (24,1-32,7)
Amazonas	30,6 (26,9-34,5)	40,2 (34,4-46,3)	20,9 (16,2-26,6)
Roraima	33,4 (30,0-36,9)	43,5 (38,8-48,4)	25,7 (22,7-29,0)
Para	38,5 (34,6-42,6)	46,4 (40,9-52,1)	31,5 (26,3-37,2)
Amapá	38,8 (35,0-42,7)	49,8 (44,8-54,8)	27,9 (23,6-32,7)
Tocantins	41,4 (36,3-46,7)	56,2 (48,2-63,9)	29,8 (24,7-35,4)
Maranhão	28,5 (24,2-33,2)	38,6 (32,3-45,2)	21,7 (17,7-26,2)
Piauí	36,0 (30,7-41,6)	48,1 (40,2-56,0)	23,5 (18,5-29,5)
Ceará	36,7 (32,3-41,3)	44,2 (38,7-49,8)	29,0 (23,8-34,8)
Rio Grande do Norte	36,2 (32,3-40,2)	47,4 (41,5-53,3)	25,9 (21,5-30,7)
Paraíba	36,7 (32,8-40,9)	49,4 (43,8-55,0)	25,7 (21,7-30,0)
Pernambuco	34,1 (29,5-38,9)	48,5 (42,0-55,1)	21,0 (16,2-26,8)
Alagoas	33,5 (28,9-38,3)	45,7 (40,3-51,2)	24,4 (18,8-30,9)
Sergipe	37,8 (33,2-42,5)	46,6 (41,3-52,1)	28,3 (22,7-34,6)
Bahia	34,7 (29,6-40,1)	43,5 (35,1-52,3)	26,0 (22,9-29,4)
Minas Gerais	38,8 (33,4-44,6)	50,7 (41,0-60,3)	28,0 (23,2-33,3)
Espírito Santo	37,7 (33,3-42,3)	44,8 (37,2-52,6)	29,5 (24,4-35,2)
Rio de Janeiro	40,7 (37,6-43,9)	51,6 (46,7-56,4)	30,8 (27,4-34,3)
São Paulo	43,0 (40,0-46,0)	52,6 (47,7-57,4)	32,3 (27,6-37,4)
Paraná	33,2 (29,6-37,0)	38,6 (33,5-44,0)	28,4 (23,9-33,3)
Santa Catarina	34,6 (28,9-40,8)	41,4 (33,7-49,6)	24,5 (19,6-30,2)
Rio Grande do Sul	32,7 (27,3-38,5)	33,3 (27,2-40,1)	31,7 (24,6-39,7)
Mato Grosso do Sul	40,0 (36,5-43,5)	47,1 (40,5-53,7)	31,5 (27,0-36,3)
Mato Grosso	40,3 (33,1-48,0)	50,9 (42,0-59,7)	29,6 (23,3-36,8)
Goiás	44,2 (40,5-47,9)	51,9 (47,0-56,8)	37,4 (33,4-41,6)
Distrito Federal	42,9 (39,3-46,6)	52,6 (46,5-58,6)	33,1 (28,2-30,2)

IC95%: intervalo de 95% de confiança.

A Tabela 3 mostra, ainda, que, entre as meninas, as maiores proporções foram observadas no Tocantins (56,2%), Rondônia (53%) e São Paulo (52,6%); já as menores, foram no Rio Grande do Sul (33,3%), Maranhão (38,6%) e Paraná (38,6%). Entre os meninos, que informaram sobre o uso por suas parceiras, Goiás (37,4%), Distrito Federal (33,1%) e São Paulo (32,3%) apresentaram as maiores proporções, enquanto Amazonas (20,9%), Pernambuco (21%), e Maranhão (21,7%) apresentaram as menores, com destaque para menor uso de contraceptivos de emergência entre os adolescentes da Região Nordeste.

Não houve diferença significativa entre as estimativas de uso de contraceptivos de emergência por adolescentes de escolas públicas e privadas. Mais de um quarto dos alunos de 13 a 15 anos e mais de um terço dos de 16 e 17 anos responderam que alguma vez na vida, elas próprias, quando meninas, ou as parceiras, quando meninos, já usaram contraceptivos de emergência, em ambos os contextos (Tabela 4).

**Tabela 4:** Proporção e intervalos de 95% de confiança (IC95%) de adolescentes escolares brasileiros que responderam que já usaram contracepção de emergência alguma vez na vida por dependência administrativa da escola, faixa etária e região do país. *Pesquisa Nacional de Saúde do Escolar* (PeNSE) 2019, Brasil.

Faixa etária (anos)/Dependência administrativa da escola	Brasil	Região				
	Total	Norte	Nordeste	Sudeste	Sul	Centro-oeste
	% (IC95%)	% (IC95%)	% (IC95%)	% (IC95%)	% (IC95%)	% (IC95%)
13 a 15						
Pública	29,9 (28,4-31,4)	31,5 (28,5-34,6)	25,7 (23,2-28,3)	33,3 (30,4-36,3)	24,6 (21,0-28,6)	33,7 (30,4-37,2)
Privada	29,0 (26,7-31,5)	32,0 (26,7-37,7)	26,1 (23,6-28,9)	30,1 (25,8-34,7)	27,4 (23,0-32,2)	32,0 (27,8-36,3)
16 e 17						
Pública	43,7 (42,1-45,4)	40,3 (37,2-43,4)	40,2 (37,5-43,0)	48,1 (45,1-51,1)	38,6 (34,1-43,2)	48,9 (45,5-52,3)
Privada	44,3 (42,4-46,3)	47,1 (44,0-50,2)	45,7 (43,0-48,4)	43,0 (39,4-46,7)	44,0 (39,8-48,4)	46,3 (42,8-49,8)

No que diz respeito aos modos de ter acesso à contracepção de emergência, mais da metade dos adolescentes de 13 a 15 anos (56,6%) e aproximadamente três quartos dos de 16 e 17 anos (74,5%) adquiriram o método em farmácias comerciais, em quase todas as regiões do país (Tabela 5). Em segundo lugar, em ordem da maior para a menor, os adolescentes relataram ter adquirido os anticoncepcionais de emergência em serviços de saúde, para a faixa etária de 13 a 15 anos, e com o parceiro sexual, para aqueles com 16 e 17 anos. Esses dados estão apresentados em um *heatmap*, no qual as cores indicam variação da proporção, as mais quentes indicam as maiores frequências de respostas (vermelho-laranja-amarelo), enquanto as cores mais frias, as menores frequências de respostas (verde-azul).

**Tabela 5:** Proporção e intervalos de 95% de confiança (IC95%) dos modos de ter acesso à contracepção de emergência por adolescentes brasileiros por idade e região do país. *Pesquisa Nacional de Saúde do Escolar* (PeNSE) 2019, Brasil.

Faixa etária (anos)/Modos de ter acesso à contracepção de emergência	Brasil	Região				
	Total	Norte	Nordeste	Sudeste	Sul	Centro-oeste
	% (IC95%)	% (IC95%)	% (IC95%)	% (IC95%)	% (IC95%)	% (IC95%)
13 a 15						
Comprou na farmácia	56,6 (53,2-59,9)	52,3 (43,2-61,2)	54,6 (49,5-59,7)	59,2 (53,0-65,1)	56,8 (47,7-65,5)	55,0 (50,0-60,0)
No serviço de saúde	15,2 (12,8-17,9)	24,4 (15,3-36,7)	19,3 (15,1-24,4)	8,9 (6,5-12,0)	20,5 (13,5-29,9)	14,0 (10,7-18,0)
Com o parceiro sexual	11,8 (9,9-14,0)	10,8 (8,2-14,1)	11,6 (8,5-15,5)	12,5 (9,0-17,2)	10,0 (5,9-16,5)	12,7 (10,1-15,7)
Com amigo ou colega	6,4 (4,9-8,2)	5,1 (2,8-9,0)	7,2 (4,9-10,5)	6,7 (4,2-10,7)	2,3 (0,9-5,6)	9,4 (6,5-13,4)
Com mãe, pai ou responsável	5,9 (4,5-7,7)	3,7 (2,2-6,2)	4,9 (3,2-7,4)	6,6 (4,0-10,7)	8,0 (4,7-13,3)	5,7 (3,9-8,3)
Com outra pessoa ou outro modo	4,2 (0,3-5,6)	3,8 (2,2-6,5)	2,4 (1,5-3,9)	6,0 (4,0-09,0)	02,4 (1,0-5,5)	3,2 (1,9-5,4)
16 e 17						
Comprou na farmácia	74,5 (72,7-76,2)	66,3 (62,6-69,8)	72,6 (69,3-75,6)	77,3 (74,2-80,2)	75,3 (69,4-80,3)	74,9 (71,0-78,4)
No serviço de saúde	9,1 (7,9-10,5)	11,9 (9,2-15,3)	10,8 (8,9-13,1)	7,5 (5,4-10,3)	9,4 (6,7-13,1)	8,0 (5,8-10,9)
Com o parceiro sexual	9,7 (8,6-10,8)	14,6 (11,3-18,6)	10,2 (8,4-12,3)	8,9 (7,2-11,0)	7,0 (4,6-10,3)	9,6 (7,6-11,9)
Com amigo ou colega	2,8 (2,2-3,6)	3,0 (2,1-4,1)	3,4 (2,3-5,0)	2,7 (1,7-4,2)	2,4 (1,0-5,7)	2,2 (1,3-3,6)
Com mãe, pai ou responsável	2,8 (0,2-3,6)	3,8 (2,4-6,1)	2,1 (1,3-3,3)	2,3 (1,4-3,8)	4,8 (2,8-7,9)	2,7 (1,8-4,2)
Com outra pessoa ou outro modo	1,2 (0,9-1,7)	0,5 (0,3-0,9)	1,0 (0,6-1,5)	1,3 (0,7-2,4)	1,3 (0,5-2,9)	2,7 (1,6-4,5)

Nota: as cores indicam variação da proporção, as mais quentes indicam as maiores frequências de respostas (vermelho-laranja-amarelo), enquanto as cores mais frias, as menores frequências de respostas (verde-azul).

A Tabela 6 apresenta a análise multivariada com as razões de proporções não ajustada e ajustada dos fatores associados ao uso de contraceptivos de emergência por adolescentes brasileiros. No modelo final, ajustado, manteve-se a associação entre as variáveis sexo, faixa etária, histórico de violência sexual, procura por serviço de saúde no último ano, morar com os pais e região de moradia com o uso de contraceptivos de emergência. Assim, as meninas (RP ajustada = 1,55; IC95%: 1,45-1,65), os adolescentes de 16 e 17 anos (RP ajustada = 1,45; IC95%: 1,37-1,53), aqueles que já sofreram violência sexual (RP ajustada = 1,16; IC95%: 1,08-1,24), aqueles que procuraram o serviço de saúde no último ano (RP ajustada = 1,12; IC95%: 1,06-1,18), que moram somente com o pai (RP ajustada = 1,20; IC95%: 1,08-1,34) ou só com a mãe (RP ajustada = 1,08; IC95%: 1,02-1,15) ou sem nenhum dos pais (RP ajustada = 1,14; IC95%: 1,04-1,24) e que residem na Região Centro-oeste (RP ajustada = 1,13; IC95%: 1,05-1,22) ou na Região Sudeste (RP ajustada = 1,10; IC95%: 1,02-1,19) tiveram maior chance de usar contraceptivos de emergência. Morar na Região Sul foi fator protetor ao uso de contraceptivos de emergência, ou seja, observou-se menor chance de usar (RP ajustada = 0,89; IC95%: 0,80-0,98).

**Tabela 6:** Razão de proporções (RP) não ajustada e ajustada dos fatores associados ao uso de contracepção de emergência alguma vez na vida, por adolescentes escolares brasileiros, segundo as próprias adolescentes, quando meninas, ou as parceiras, quando meninos. *Pesquisa Nacional de Saúde do Escolar* (PeNSE), 2019, Brasil.

Variáveis explicativas	Uso de contracepção de emergência	Valor de p *	Uso de contracepção de emergência	
	% ** (IC95%)		RP não ajustada (IC95%)	RP ajustada (IC95%)
Fatores individuais				
Sexo (n = 33.603)		0,0001		
Masculino	28,8 (27,4-30,2)		Referência	Referência
Feminino	47,1 (45,3-48,9)		1,64 (1,54-1,74)	1,55 (1,45-1,65)
Faixa etária (anos) (n = 33.711)		0,0001		
13 a 15	29,8 (28,5-31,2)		Referência	Referência
16 e 17	43,8 (42,3-45,3)		1,47 (1,39-1,55)	1,45 (1,37-1,53)
Raça/Cor (n = 32.980)		0,0233		
Branca	39,5 (37,4-41,7)		Referência	
Preta	35,5 (32,9-38,2)		0,89 (0,81-0,99)	
Parda	37,5 (35,8-39,1)		0,95 (0,88-1,02)	
Amarela	42,3 (37,3-47,5)		1,07 (0,93-1,23)	
Indígena	32,1 (26,3-38,6)		0,81 (0,66-1,00)	
Gravidez prévia (n = 16.123)		0,0027		
Não	46,6 (44,7-48,4)		Referência	
Sim	54,8 (49,4-60,0)		1,18 (1,07-1,30)	
Histórico de violência doméstica (n = 33.444)		0,0054		
Não	37,0 (35,8-38,3)		Referência	
Sim	40,2 (38,2-42,2)		1,04 (1,01-1,07)	
Histórico de importunação sexual (n = 33.711)		0,0001		
Não	36,0 (34,8-37,3)		Referência	
Sim	44,3 (41,9-46,6)		1,23 (1,15-1,31)	
Histórico de violência sexual (n = 33.416)		0,0001		
Não	36,5 (35,4-37,7)		Referência	Referência
Sim	48,1 (44,9-51,3)		1,32 (1,23-1,41)	1,16 (1,08-1,24)
Procura por serviço de saúde no último ano (n = 33.419)		0,0001		
Não	34,1 (32,4-35,9)		Referência	Referência
Sim	40,4 (39,1-41,7)		1,18 (1,12-1,25)	1,12 (1,06-1,18)
Se vacinou contra o HPV (n = 27.082)		0,2197		
Não	37,5 (34,9-40,1)		Referência	
Sim	39,2 (37,8-40,7)		1,05 (0,97-1,13)	
Fatores familiares				
Escolaridade da mãe (n = 29.421)		0,0001		
Não estudou	30,2 (25,4-35,4)		Referência	
Ensino Fundamental	36,9 (34,8-39,0)		1,14 (1,07-1,21)	
Ensino Médio	41,4 (39,5-41,4)		1,18 (1,05-1,32)	
Ensino Superior	40,1 (38,1-42,0)		1,26 (1,14-1,39)	
Mora com os pais (n = 33.683)		0,0001		
Com ambos os pais	34,9 (33,5-36,3)		Referência	Referência
Somente com a mãe	39,6 (37,7-41,5)		0,94 (0,92-0,97)	1,08 (1,02-1,15)
Somente com o pai	41,1 (37,0-45,2)		0,96 (0,92-1,01)	1,20 (1,08-1,34)
Com nenhum dos pais	43,8 (39,9-47,8)		0,92 (0,89-0,96)	1,14 (1,04-1,24)
Supervisão dos pais (n = 33.513)		0,0001		
0 (nenhuma)	44,4 (37,7-51,2)		Referência	
1	40,2 (37,5-43,0)		0,91 (0,77-1,06)	
2	39,3 (37,4-41,2)		0,89 (0,76-1,03)	
3	37,8 (36,1-39,6)		0,85 (0,73-1,00)	
4 (intensa)	33,6 (31,6-35,6)		0,76 (0,64-0,90)	
Fatores comunitários e políticos				
Região de moradia (n = 33.711)		0,0001		
Norte	36,6 (34,5-38,8)		Referência	Referência
Nordeste	34,3 (32,3-36,3)		0,94 (0,86-1,02)	0,94 (0,87-1,02)
Sudeste	41,4 (39,3-43,5)		1,13 (1,05-1,22)	1,10 (1,02-1,19)
Sul	33,4 (30,5-36,4)		0,91 (0,82-1,01)	0,89 (0,80-0,98)
Centro-oeste	42,3 (39,8-44,8)		1,16 (1,06-1,26)	1,13 (1,05-1,22)
Situação da área de moradia (n = 33.711)		0,0001		
Rural	26,9 (22,9-31,3)		Referência	
Urbana	38,7 (37,6-39,9)		1,44 (1,23-1,69)	
Dependência administrativa da escola (n = 33.711)		0,9473		
Pública	37,9 (36,7-39,2)		Referência	
Privada	37,8 (36,3-39,4)		1,00 (0,95-1,05)	
Orientação sobre prevenção de gravidez na escola (n = 33.662)		0,6582		
Não	38,3 (36,2-40,4)		Referência	
Sim	37,8 (36,5-39,0)		0,99 (0,93-1,05)	
Orientação na escola sobre prevenção de IST (n = 33.656)		0,9060		
Não	38,0 (35,2-41,0)		Referência	
Sim	37,9 (36,6-39,1)		1,0 (0,92-1,08)	
Orientação na escola sobre acesso a preservativo gratuito (n = 33.647)		0,0783		
Não	36,0 (33,8-38,3)		Referência	
Sim	38,3 (37,0-40,0)		1,06 (0,99-1,14)	

IC95%: intervalo de 95% de confiança; IST: infecções sexualmente transmissíveis.

* Teste qui-quadrado de Pearson;

** Estimativa populacional.

## Discussão

Cerca de um terço dos adolescentes de 13 a 15 anos e quase metade entre 16 e 17 anos usaram a contracepção de emergência no país. A maior proporção de uso de contraceptivos de emergência foi reportada pelas meninas, independentemente da faixa etária e em todas as regiões do país. Observou-se variação da proporção do uso de contraceptivos de emergência entre as UFs brasileiras, independentemente da região, com alguns possíveis padrões como o maior uso nas UFs do Centro-oeste e Sudeste e menor uso nas UFs do Nordeste e Sul. Entre os adolescentes mais velhos, verificou-se maior proporção de uso de contraceptivos de emergência entre aqueles das escolas privadas, em todas as regiões do país, exceto no Sudeste e Centro-oeste, em que a maior taxa de uso está entre os alunos das escolas públicas. Em relação aos modos de ter acesso à contracepção de emergência, as farmácias comerciais representaram a principal maneira de aquisição, independentemente da faixa etária e da região, seguido dos serviços públicos de saúde, entre os adolescentes de 13 a 15 anos, e com os parceiros sexuais entre os de 16 e 17 anos.

Esses achados se assemelham àqueles de estudos prévios ^6^ ^,^ ^18^, no sentido do uso mais prevalente de contraceptivos de emergência, reforçando o cenário de comportamentos sexuais que colocam em risco a saúde dos adolescentes. Ao mesmo tempo, esse método é eficaz em proteger contra uma gravidez não planejada e é um direito em situações em que os contraceptivos de uso regular ou métodos de barreira não foram utilizados ^22^. Ainda, a magnitude do uso pode indicar o não uso de contracepção regular, e, consequentemente, maior exposição a gestações não planejadas, além de riscos de contrair IST. As evidências de aumento das IST, como a sífilis, nessa faixa etária ^30^ e a manutenção das taxas de gravidez na adolescência no país ^3^ podem reforçar essa hipótese. Portanto, pode-se inferir fragilidade de políticas e ações programáticas efetivas e consubstanciadas de contracepção para esse grupo, que poderia ser endossado pelo retrocesso político e programático observado no campo da saúde sexual e reprodutiva no país ^17^ ^,^ ^31^, com quadro mais dramático para os adolescentes ^17^.

O uso de contraceptivos de emergência pode também elucidar a falta de preservativos no momento da relação sexual, a não confiança em outros métodos ou a falta de acesso a eles, rompimento ou retenção do preservativo e mesmo o uso inadequado da contracepção de rotina ^5^ ^,^ ^32^ ^,^ ^33^, o que reforça as fragilidades programáticas. Outro aspecto que esses resultados podem indicar é acesso mais dificultado, principalmente aos mais jovens, o que também implica em menor chance de obter informações e garantia dos direitos sexuais e reprodutivos nos serviços de saúde.

Nossos achados demonstram que os fatores individuais como o uso ter sido relatado mais frequentemente pelas meninas, por jovens da faixa etária de 16 e 17 anos, com histórico de violência sexual e que procuraram por serviço de saúde no último ano, estavam positivamente associados ao uso de contraceptivos de emergência; assim como os fatores familiares de residir somente com pai ou só com a mãe ou com nenhum dos pais. Fatores comunitários, como a região de residência, também apresentam associação com o uso, visto que os adolescentes do Centro-oeste e Sudeste são os que mais utilizam e os da Região Sul são os que menos utilizam contraceptivos de emergência. Esses fatores mostram quem está conseguindo acessar mais esse método, tanto no âmbito da informação quanto no âmbito de seu uso propriamente dito.

Em relação aos fatores individuais, se destacaram o sexo, a faixa etária e a procura por serviço de saúde no último ano. Os achados relativos ao sexo poderiam ser explicados pelas desigualdades de gênero no âmbito da contracepção. Ou seja, os meninos reportaram em menor proporção o uso por suas parceiras, o que reforça um padrão social no qual a responsabilidade sobre a prevenção da gravidez diz respeito às mulheres, o que já aparece entre os adolescentes, elucidando o desafio de incluir os meninos nas abordagens em relação à prevenção de gravidez na adolescência ^17^. Esse resultado corrobora os de outros estudos ^5^ ^,^ ^20^ ^,^ ^33^, que muitas vezes não incluem os meninos em questões relacionadas à contracepção. Tal fato elucida a distância da meta de igualdade de gênero e que ações estruturais e intersetoriais seriam necessárias para fomentar a mudança desse cenário.

Quanto à idade, o maior uso de contraceptivos de emergência foi observado entre os adolescentes médios, quase o dobro do uso entre os mais jovens, o que poderia ser explicado pela maior orientação sobre o uso de métodos contraceptivos ^1^ ^,^ ^6^, bem como maior facilidade no acesso ao método ^9^ ^,^ ^34^, refletindo maior capacidade cognitiva e de responsabilidade na adolescência média em relação à inicial ^35^ ^,^ ^36^. E aqueles que usaram o serviço de saúde no último ano também tiveram maior chance de usar contraceptivos de emergência, o que poderia ser explicado por maior acesso daqueles que usam os serviços, tanto à informação quanto ao próprio método.

Por outro lado, a maior parte dos adolescentes adquiriu o método diretamente nas farmácias comerciais, o que pode estar relacionado ao atendimento rápido, a não necessidade de receitas, de consultas com profissionais de saúde, o que facilita o acesso ^32^ ^,^ ^34^ e garante o direito ao uso. No entanto, pode ser um evento sentinela da falta de acesso dos adolescentes a serviços de saúde qualificados para atendimento das suas demandas. Outro problema é que, ao acessar a farmácia, perde-se a janela de oportunidade de aconselhamento e educação em saúde acerca da saúde sexual e reprodutiva e de acesso aos métodos para uso contínuo.

Adolescentes que já sofreram violência sexual tiveram mais respostas sim sobre o uso de contraceptivos de emergência do que aqueles que não sofreram esse tipo de violência. Isso pode se dar devido a protocolos de atendimento que orientam a prescrição e disponibilização da contracepção de emergência às vítimas de violência ^37^. A violência pode ser capaz de gerar diversos transtornos na vida do adolescente, que se prolongam até a vida adulta, como sintomas depressivos e ansiosos, redução da qualidade de vida, maior uso de tabaco e outras drogas ^38^, podendo ainda gerar inseguranças contraceptivas.

O modelo multivariado mostrou que residir com os pais se associou ao uso de contraceptivos de emergência. Assim, morar com ambos os pais se mostrou fator protetor em relação ao uso, pois quando há uma boa estrutura familiar e maior proximidade com os adolescentes, maior a chance de redução de comportamentos sexuais de risco ^39^. Outro estudo mostrou que quando há comunicação entre pais e adolescentes sobre saúde sexual e reprodutiva, aumenta o conhecimento dos adolescentes em relação à gravidez, acesso a preservativos e a outros métodos contraceptivos ^40^. Estudo que relacionou a supervisão dos pais com os comportamentos sexuais de risco também mostrou que adolescentes que apresentam maior supervisão parental fazem maior uso de métodos contraceptivos e, desse modo, ficam menos expostos a uma gravidez ^25^ ^,^ ^28^. Neste estudo, apenas na análise univariada a maior supervisão se associou a uma redução de 24% na chance de uso de contraceptivos de emergência ao se comparar com os adolescentes com escore zero de supervisão. Essa relação não permaneceu no modelo final, apesar de estudos prévios terem demonstrado essa associação inclusive com outros comportamentos em saúde ^25^ ^,^ ^26^ ^,^ ^28^, o que pode ser explicado a partir da significância de outros fatores familiares presentes no modelo estudado. Isso pode ser confirmado pela análise da relação entre as variáveis explicativas avaliada previamente à modelagem e mostrou que 58% dos jovens que moravam com ambos os pais tinham maior supervisão com significância estatística (p < 0,0001).

Em relação aos fatores comunitários, as diferenças de acordo com a região em que o adolescente vive, podem estar relacionadas a aspectos sociopolíticos, culturais e de acesso mais locais à contracepção. Em outros grupos populacionais, como mulheres em idade reprodutiva, verificou-se que há diferenças entre as regiões do país em relação ao uso de métodos contraceptivos ^31^ e de contracepção de emergência ^9^. Essa heterogeneidade evidencia fragilidades das políticas públicas para garantia dos direitos sexuais e reprodutivos. A Região Sul, que apresentou menor uso de contraceptivos de emergência, é a região com maior uso de anticoncepcionais orais pelos adolescentes, pois em 2019 a proporção do uso entre aqueles de 13 a 15 anos foi de 58,9% e nos de 16 e 17 anos foi superior a 70% ^1^. Assim, o menor uso pode não representar menor acesso e sim melhor cenário em relação à contracepção mais consistente e regular.

Uma das limitações deste estudo consiste na inclusão apenas de adolescentes que estavam regularmente matriculados em escola pública ou privada no ano de 2019. Sabe-se que, em 2019, 10,8% da população dessa faixa etária não estava matriculada em nenhuma escola ^41^. Essa população, se incluída no estudo, poderia revelar cenários diferentes, pois a falta de acesso à escola poderia representar também falta de acesso a outros bens e serviços, incluindo informações e uso de contraceptivos de emergência. Assim, esses jovens podem também fazer maior uso de contraceptivos de emergência em detrimento de uma contracepção regular, configurando-se em um viés conservador. Outra limitação foi o uso de medida proxy de supervisão parental, não abordando todos os aspectos relacionados à supervisão. Tem-se ainda a impossibilidade de estimar a prevalência do uso de contraceptivos de emergência, porque foram considerados em risco apenas aqueles que relataram ter iniciado a vida sexual, alterando a estimativa para esse subgrupo populacional. Porém, mesmo com o recorte, trata-se de amostra aleatória, abrangente, com representantes de todo o país.

Este estudo avança por mostrar por meio de uma grande amostra aleatória e em âmbito nacional, de forma inédita, estimativas de uso de contraceptivos de emergência no Brasil pelos adolescentes de 13 a 17 anos, e principalmente os fatores individuais, familiares e comunitários associados ao seu uso, conforme modelo sociológico e uso de regressão binomial negativa, apropriada para analisar desfechos prevalentes, por se constituir em método de estimação de parâmetros mais robustos ^42^ ^,^ ^43^. Esses aspectos metodológicos permitem maior poder amostral para detectar associações que poderiam não ser detectadas em estudos menores, vantagem significativa para testar nossa hipótese e aumentar a validade das associações encontradas.

Assim, os fatores associados ao uso de contracepção precisam ser considerados ao se repensar as ações político-programáticas voltadas à promoção do sexo seguro a esse grupo no país. Os adolescentes mais jovens têm diferenciais importantes a serem observados em relação aos mais velhos, os meninos precisam ser incluídos, ações com famílias e pais podem ser relevantes e o acesso precisa ser facilitado e qualificado. Algumas desigualdades também precisam ser observadas na oferta desse serviço, buscando equidade daqueles mais alijados. Iniciativas de sucesso têm sido reportadas, com destaque para as intervenções educativas em sexualidade que tem como princípio modelos teóricos de mudança de comportamento, metodologias ativas, que abrangem ambos os sexos, com facilitadores capacitados e que tenham mais tempo de intervenção ^44^. A persistência da desigualdade de gênero que aparece aqui em jovens de 13 a 17 anos também revela o quão distantes o país se encontra dessa meta dos Objetivos de Desenvolvimento Sustentável (ODS). Trata-se de um desafio em cenário tão conservador marcado por questões morais e religiosas, dificultando cada vez mais a garantia dos direitos sexuais e reprodutivos dos adolescentes brasileiros.

## Conclusão

O uso de contraceptivos de emergência reportado pelas meninas, por adolescentes mais velhos, com histórico de violência sexual, que procuraram o serviço de saúde no último ano, que vivem somente com o pai ou somente com a mãe ou sem nenhum dos pais apresentaram associação com maior uso de contraceptivos de emergência. Ou seja, os jovens que conseguem acessar ou têm mais condições de acessar os serviços de saúde, acessam mais o método, revelando inequidades. Além disso, a saúde sexual e reprodutiva segue marcada por questões de gênero, com menor participação dos meninos. Esses achados indicam possíveis falhas no acesso e utilização de outros métodos contraceptivos de forma mais consistente, bem como insegurança contraceptiva dos adolescentes.
